# Metabolic syndrome and lifestyle factors among type 2 diabetes mellitus patients in Dessie Referral Hospital, Amhara region, Ethiopia

**DOI:** 10.1371/journal.pone.0241432

**Published:** 2020-11-02

**Authors:** Aregash Abebayehu Zerga, Afework Mulugeta Bezabih

**Affiliations:** 1 Department of Nutrition, College of Medicine and Health Sciences, Wollo University, Dessie, Ethiopia; 2 Department of Nutrition and Dietetics, College of Health Sciences, Mekelle University, Mekelle, Ethiopia; Università degli Studi di Milano, ITALY

## Abstract

**Background:**

The prevalence of metabolic syndrome is rising at an alarming rate and more common among Type 2 Diabetes Mellitus patients in the world. The risk for cardiovascular disease is greater among individuals who have a combination of Type 2 Diabetes Mellitus and metabolic syndrome compared to those who have either alone.

**Objective:**

To assess the proportion of metabolic syndrome and lifestyle factors among Type 2 Diabetes Mellitus Patients in Dessie Referral Hospital, Amhara Region, Ethiopia.

**Methods:**

A hospital-based cross-sectional study was conducted from February to March 2017 among 343 randomly selected Type 2 Diabetes Mellitus patients. Three definitions of Metabolic syndrome were considered. Multivariable logistic regression analysis was conducted to identify factors associated with metabolic syndrome. Adjusted odds ratio (AOR) with 95% confidence intervals (CI) were reported to show the strength of association. Statistical significance was declared at P-value < 0.05.

**Result:**

The proportion of metabolic syndrome was 50.3%, 59.4% and 64.5% according to 2005 International Diabetes Federation, revised ATP III and 2009 harmonized criteria, respectively. Being female (AOR = 2.43; 95% CI = 1.40, 4.21), consumption of red meat (AOR = 2.61; 95% CI = 1.28, 5.33), sedentary leisure time activity (AOR = 2.65; 95% CI = 1.47, 4.78), coffee intake (AOR = 0.43; 95% CI = 0.21, 0.86), BMI ≥ 25 kg/m^2^ (AOR = 9.59; 95% CI = 4.98, 18.47), 40–49 years of age (AOR = 2.74, 95% CI = (1.02, 7.37), 50–59 years of age (AOR = 4.22; 95% CI = 1.60, 11.11) and ≥70 years of age (AOR = 4.51, 95% CI = 1.44, 14.15) were significantly associated with metabolic syndrome.

**Conclusion and recommendation:**

The proportion of metabolic syndrome was high among Type 2 Diabetes Mellitus patients. Overweight and obesity, being female, age of respondent, intake of coffee, regular red meat consumption, and sedentary leisure-time activity were factors associated with metabolic syndrome. Counseling of Type 2 Diabetes Mellitus patients on the need for spending leisure time with activities, intake of coffee, control of body weight, and avoidance of regular red meat consumption is recommended.

## Introduction

Since 1923, different expert groups put forward several definitions for metabolic syndrome (MetS) [1–4]. Recently, MetS is defined as the presence of at least three of the five interrelated risk factors (triglyceride (TG) > 150 milligram per deciliter (mg/dl), high density lipoprotein (HDL) < 40 mg/dl for men and < 50 mg/dl for women, blood pressure (BP) > 130/ 85 millimeter mercury (mmHg), fasting blood glucose (FBG) > 100 mg/dl and waist circumference (WC) >102 cm for men and > 88 cm for women) [5].

Globally, the prevalence of MetS is rising at an alarming rate and more common among Type 2 Diabetes Mellitus (T2DM) patients [6]. It was estimated that 25% of the general population and 70% to 80% of T2DM patients had MetS in the world [7]. Based on a systematic review in Sub-Saharan Africa, the prevalence of MetS among T2DM patients was 59.62% [8]. In Ethiopia, the prevalence of MetS among T2DM patients reached up to 70.1% with different diagnostic criteria of MetS [9–11].

Individuals with MetS were three and two times more likely to have a higher risk of heart attack and cardiovascular diseases (CVD) respectively as compared to individuals without MetS [12, 13]. Approximately 6–7% of all-cause mortality and 12–17% of CVD was attributable to MetS [13]. Further, people with MetS were more vulnerable to a variety of diseases such as fatty liver, sleep disorders, polycystic ovary syndrome, cholesterol gallstones, asthma, and malignancies [3]. It is recognized that the risk of CVD development is greater among individuals who have a combination of T2DM and MetS compared to those who had either alone [14].

A high prevalence of MetS could be due to a rapid nutrition transition such as high-calorie intake, high fat consumption, and low consumption of dietary fiber foods, as well as behavioral factors like sedentary lifestyles, increase in tobacco use, and excessive alcohol consumption [15]. However, studies on the prevalence of MetS and associated lifestyle factors among T2DM patients are not available in the study area. Therefore, this study examines it among T2DM patients in the Dessie Referral Hospital.

## Methods and materials

### Study area, period and design

A hospital-based cross-sectional study was conducted in Dessie Referral Hospital from February to March 2017. The Hospital is located in Dessie town and it is 401 km to the north of Addis Ababa, the capital of Ethiopia. The Diabetes clinic of the hospital gives services on all working days (Monday to Friday), and on average it has been visited by twenty-seven T2DM patients per day.

### Study population and eligibility criteria

All T2DM patients with age ≥ 30 years who had follow-up in Dessie referral hospital during the data collection period were eligible for this study. Patients who were unable to respond due to severe illness, who had physical deformity, and ate breakfast were excluded from the study.

### Sample size determination and sampling technique

The sample size was determined using a single population proportion formula, $n={\left(Z1-\frac{\alpha }{2}\right)}^{2}p(1-p)/d^{2}$. Where: n = sample size, p = proportion of MetS among T2DM patients in Sub-Saharan Africa (71.7%) [16], Z_1-α/2_ = standard normal deviation at 95% confidence interval (1.96), and d = margin of error (5%). The calculated sample size was 312. By adding 10% for non-response rate the final sample size (n) was **343**. By taking the register as a sampling frame, simple random sampling technique was employed to select study participants.

### Operational/standard definitions

#### Dietary Diversity Score (DDS)

Individuals DDS were grouped into low (≤ 3 food groups), medium (4 to 6 food groups), and high (7 to 9 food groups) [17].

#### Knowledge

Seven multiple-choice questions about MetS with a total of 17 responses (Cronbach’s alpha = 0.82) were asked, and summed. Then individuals knowledge were grouped into low (≤ 50%), fair (51 to 80%), and good (>80%) [18].

#### Physical activity

Individuals who did moderate-intensity activity for at least 30 minutes per day on at least five days per week were considered as physically active [19].

#### Adequate sleep

Participants who sleep 7 to 8 hours per day without difficulty initiating and maintaining sleep are grouped as having adequate sleep [12].

#### Metabolic syndrome

Three definitions of MetS were considered. However, a definition that had better agreement with the rest two definitions, after calculating Kappa statistics, was used in this study to run the regression analysis.

#### The Revised National Cholesterol Education Program: Third adult treatment panel (ATP III) definition

MetS is the group of at least 3 of the following 5 risk factors: WC >102 for men and >88 cm for women, BP >130/85 mmHg or on treatment, FBG ≥ 110 mg/dl or on treatment, TG ≥ 150 mg/dl or on treatment and HDL < 40mg/dl for men and < 50mg/dl for women [2].

#### International Diabetes Federation (IDF) definition

MetS is WC > 80cm for women, > 94 cm for men plus 2 or more the factors (BP ≥ 130/85 mmHg or on treatment, TG ≥ 150 mg/dl or on treatment HDL < 40 mg/dl for men and < 50 mg/dl for women or on treatment, and FBG ≥ 100 mg/dl) [4].

#### The 2009 harmonized criteria

MetS is a cluster of three or more of the 5 interrelated risk factors (TG ≥ 150 mg/dl, HDL < 40 mg/dl for men and < 50mg/dl for women, BP > 130/85 mmHg, FBG ≥ 100 mg/dl, and WC >102 cm for men and >88 cm for women) [5].

#### Body mass index (BMI)

It is classified into underweight (<18.5 kilogram per meter square (kg/m^2^)), normal weight (18.5–24.9kg/m^2^), overweight (25–29.9 kg/m^2^) and obese (>30 kg/m^2^) [20].

### Data collection tools and measurements

Data were collected by the World Health Organization STEPwise approach for non-communicable disease surveillance [19]. Data about socio-demographic, medication, behavioral, and dietary factors were collected by a structured questionnaire. Height was measured in a standing position by a height measuring board on barefoot. Weight was measured using SECA weight measuring scale. WC was measured by a normal tension measuring tape at the midpoint of the inferior margin of the last rib and the iliac crest at the end of expiration [21]. Each anthropometric measurement was recorded to the nearest 0.1cm (kg for weight). Blood pressure was measured by sphygmomanometer from the right arm after participants rested for 5 minutes. Data were collected by 4 trained BSc nurses.

For the biochemical analysis, 5 milliliters fasting venous blood was taken from each study participant. After allowing the formation of clots by staying the whole blood for 30 minutes, the serum was separated by centrifuging the clotted blood at 3,000 revolutions per minute. FBG and lipid profiles were determined from serum blood using the DIRUI CS-T240 Auto chemistry analyzer by laboratory technologists following the standard operating procedures. Additionally, the intake of medication was assessed from the patient card.

### Data quality control

The questionnaire was translated to Amharic and back to English for consistency. Data collectors and a supervisor were trained on the data collection process. The questionnaire was pre-tested on 5% (17) of the sample size at Boru-Meda General Hospital. The completeness of the questionnaire and correctness of the measuring procedure was monitored daily by a supervisor. Weight measurement scale was standardized before each measurement by placing 2 kg iron bars. Each anthropometric measurement was taken two times, and the measurement was repeated if the difference between the two measurements exceeds 1 cm (100g for weight).

To keep the quality of biochemical assessment, training was given for laboratory professionals using standard operating procedures, and chemicals were monitored for expiry date and proper storage. Calibration was done when; new reagent was introduced, control test not passed and unexpected value produced.

### Data management and analysis

The collected data were entered into Epi Data version 3.1 and exported to statistical package for social science (SPSS) version 20.0 for analysis. Outcome variable was dichotomized into 1 = with MetS and 0 = without MetS. The normality of continuous data was checked with histograms. Descriptive statistics were computed. Normally distributed variables were reported with mean and standard deviation., and not-normally distributed variables were reported with median and interquartile range. Categorical variables were reported with frequency and percentiles. The outcome variable was reported in percent and 95% CI. Bi-variable binary logistic regression was computed and variables with p-value <0.2 were transferred to a multi-variable logistic regression model using a backward method. Multicollinearity between independent variables was checked with standard error, and model fitness was assessed using Hosmer and Lemeshow goodness of fit test. Adjusted odds ratio (AOR) with 95% confidence intervals (CI) were reported to show the strength of association. Statistical significance was set at p-value <0.05.

### Ethical consideration

Ethical approval was obtained from the Institutional Review Committee of Mekelle University Health Science College. Written permission letter was gained from Dessie Town Health Office and Dessie Referral Hospital. Written informed consent was taken from each respondent after the purpose and objectives of the study had been informed. Participants were informed that involvement in the study was voluntary. Confidentiality and privacy were kept. Counseling was given based on an individual's result.

## Results

### Socio-demographic information

A total of 330 respondents were participated, making a response rate of 96%. Nearly half (48.5%) of the study participants were females. The age of participants was ranged from 30 to 84 years old. Ninety-six (29.1%) of the study participants were found in the age range of 50 to 59 years. Only 70 (21.2%) of the study participants had diploma and higher levels of education. Nearly one fourth (24.5%) and 75 (22.7%) of the study participants were housewives and rural residents, respectively (Table 1).

**Table 1:** Socio-demographic information of type 2 diabetes mellitus patients from Dessie Referral Hospital, Amhara region, Ethiopia, 2017 (n = 330).

Variables	Categories	With MetS = 196	Without MetS = 134	Total (percent)
Sex	Male	84 (42.9)	86 (64.2)	170 (51.5)
	Female	112(57.1)	48 (35.8)	160 (48.5)
Age group	30–39	14 (7.2)	24 (17.9)	38 (11.5)
	40–49	45(22.9)	33(24.6)	78 (23.7)
	50–59	66(33.7)	30 (22.4)	96 (29.1)
	60–69	43(21.9)	36 (26.9)	79 (23.9)
	> = 70	28(14.3)	11(8.2)	39 (11.8)
Religion	Orthodox	96 (49)	42 (31.3)	138 (41.8)
	Muslim	100 (51)	92 (68.7)	192 (58.2)
Educational status	Cannot read and write	74 (37.8)	61 (45.5)	135 (40.9)
	Primary education	48 (24.5)	31 (23.1)	79 (23.9)
	Secondary education	29 (14.8)	17 (12.7)	46 (13.9)
	Diploma and above	45 (22.9)	25 (18.7)	70 (21.2)
Marital status	Single	8 (4.1)	6 (4.5)	14 (4.2)
	Married	130 (66.3)	96 (71.6)	226 (68.5)
	Divorced	20 (10.2)	18 (13.4)	38 (11.5)
	Widowed	38 (19.4)	14 (10.5)	52 (15.8)
Occupational status	Housewife	54 (27.6)	27 (20.2)	81(24.5)
	Government employed	57 (29.1)	29 (21.7)	86 (26.1)
	Farmer	23 (11.7)	44 (32.8)	67 (20.3)
	Merchant	38 (19.4)	18 (13.4)	56 (17.0)
	Retire	20 (10.2)	7 (5.2)	27 (8.2)
	Other	4 (2.0)	9 (6.7)	13 (3.9)
Place of residence	Urban	170 (86.7)	85 (63.4)	255 (77.3)
	Rural	26 (13.3)	49 (36.6)	75 (22.7)

Where; other = daily laborer and guardian.

### Medication and behavioral factors

The median length of medication for sugar level was 5 ±7 years and ranged from 6 months to 31 years. Ninety-two (27.9%) study participants had a family history for Diabetes or Hypertension. One hundred twenty-seven (38.5%) and 54 (16.4%) of the study participants had taken antihypertensive and lipid-lowering treatment, respectively. Six (1.8%) of the study participants were current smokers. Chat chewing and alcohol consumption were practiced by 132 (40%) and 88 (26.7%) of study participants, respectively. Only 50 (15.2%) of participants had good knowledge about the causes and prevention methods of MetS (Table 2).

**Table 2:** Medication and behavioral factors of type 2 diabetes mellitus patients from Dessie Referral Hospital, Amhara region, Ethiopia, 2017 (n = 330).

Variables	Categories	Frequency	Percent
Family history for chronic disease	Yes	92	27.9
	No	238	72.1
Type of medication used for T2DM	Tablet	217	65.8
	Insulin	108	32.7
	Both	5	1.5
Taking treatment for hypertension	Yes	127	38.5
	No	203	61.5
Taking lipid-lowering treatment	Yes	54	16.4
	No	276	83.6
Smoking	Current smoker	6	1.8
	Past smoker	35	10.6
	Never smoke	289	87.6
Khat chewing	Yes	132	39.7
	No	198	60.3
Drinking alcohol	Yes	88	26.7
	No	242	73.3
Physical activity	Yes	28	8.5
	No	302	91.5
Ways of spending leisure time	With sedentary activities	214	64.8
	Walking, cycling or recreational sports	116	35.2
Knowledge about MetS	Poor knowledge	144	43.6
	Fair knowledge	136	41.2
	Good knowledge	50	15.2
Sleep pattern	Inadequate	159	48.2
	Adequate	171	51.8
Presence of stress	Yes	78	23.6
	No	252	76.4

### Dietary patterns of the study participants

Almost none (0.6%) of study participants had a meal plan. One-fourth (74.8%) of participants had a meal frequency of > 3 times per day. None of the study participants had high DDS. Palm oil was consumed by 154 (46.7%) of participants. Enjera and Shiro were the staple diets for all of the study participants. Even though the recommended intake of fruits and vegetables is ≥ 5 times per day, none of the study participants had fulfilled this recommendation. The majority (77.9%) of the study participants had consumed coffee per day (Table 3).

**Table 3:** Dietary pattern of type 2 diabetes mellitus patients in Dessie Referral Hospital, Amhara region, Ethiopia, 2017.

Variables	Categories	Frequency	Percent
Meal frequency per day	≤3	247	74.8
	>3	83	25.2
Eating style	Irregular eater	99	30
	Time constraints	231	70
Type of oil used for cooking	Vegetable oil	146	44.2
	Palm oil	154	46.7
	Both	30	9.1
DDS	Low (≤3)	218	66.1
	Medium (4–6)	112	33.9
Frequency of eating vegetable	Daily	42	12.7
	Weekly	230	69.7
	Didn't take	58	17.6
Frequency of eating fruit	Daily	18	5.5
	Weekly	148	44.8
	Didn't take	164	49.7
Frequency of taking coffee	Daily	257	77.9
	Didn't take	73	22.1
Frequency of eating red meat	Daily to weekly	68	20.6
	Didn't take	262	79.4
Frequency of consuming milk and milk products	Daily	73	22.1
	Weekly	67	20.3
	Didn't take	190	57.6
Frequency of eating processed grain (pasta, macaroni, white bread)	Daily	44	13.4
	Weekly	143	43.3
	Didn't take	143	43.3
Frequency of eating chicken	Weekly	7	2.1
	Didn't take	323	97.9
Frequency of eating egg	Daily/weekly	80	24.2
	Didn't take	250	75.8
Frequency of taking fried foods	Weekly	50	15.2
	Monthly	40	12.1
	Didn’t take	240	72.7
Frequency of taking sugar and sweet	Daily/weekly	28	8.2
	Didn't take	303	91.8

### Anthropometric and biochemical measurements

**Fig 1:**
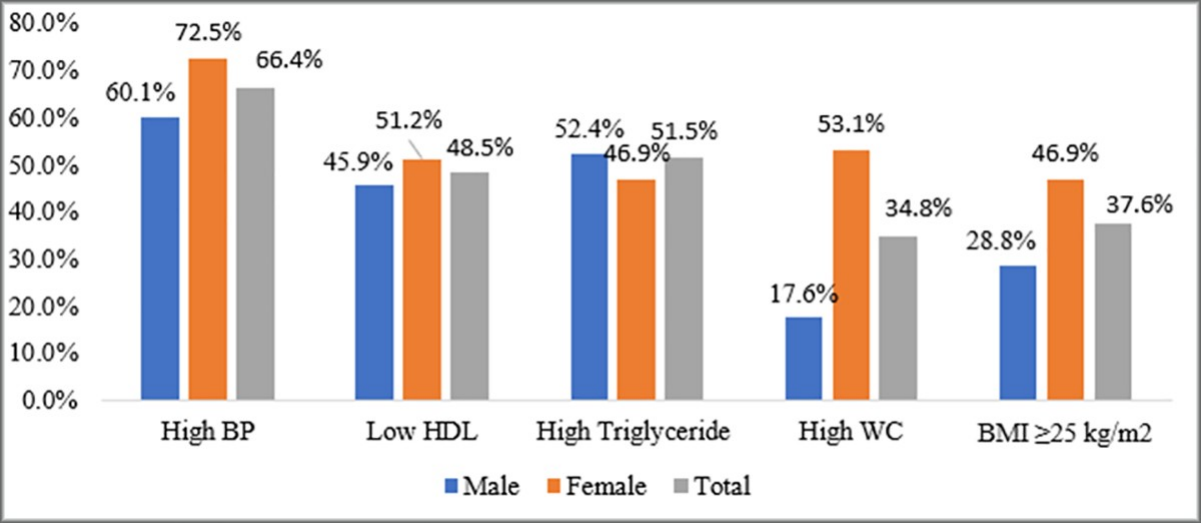
Anthropometric and biochemical measurements of T2DM patients from Dessie Referral Hospital, Amhara region, Ethiopia (N = 330).

One hundred fifteen (34.8%) of the study participants had central obesity. Almost a quarter (26.4%) of the study participants were overweight, 37 (11.2%) were obese and 18 (5.5%) were underweight. The median blood glucose level of participants was 131±76 mg/dl. Nearly half (48.5%) of the entire sample had low HDL cholesterol. The mean total cholesterol value was 189.2 mg/dl ± 47.4 mg/dl (Fig 1).

### Frequency of metabolic syndrome

The proportion of MetS with its 95% CI was 50.3% (44.8%, 55.5%), 59.4% (53.6 to 64.5) and 64.5% (59.7%, 70.2%) according to 2005 IDF, revised ATP III and 2009 harmonized definitions respectively. A very good agreement had been observed between the definition of revised ATP III and harmonized definition (Kappa = 0.89). From the total participants having MetS according to 2009 harmonized definition criteria, 92% (196/213) meets ATP III criteria. On the other hand, the IDF definition had a moderate agreement with both the ATP III and the harmonized definition with kappa (k) = 0.44 and 0.43, respectively (Table 4). So, the revised ATP III definition was used in this study.

**Table 4:** Frequency of metabolic syndrome and agreement between different definitions among type 2 diabetes mellitus patients from Dessie Referral Hospital, Amhara region, Ethiopia, 2017 (n = 330).

MetS with revised ATP III criteria		MetS by the 2009 harmonized criteria			Kappa
		Present	Absent	Total	0.89
	Present	196	0	196	
	Absent	17	117	134	
	Total	213	117	330	
MetS with IDF criteria		MetS by the ATP III criteria			0.44
		Present	Absent	Total	
	Present	135	31	166	
	Absent	61	103	164	
	Total	196	134	330	
MetS with IDF criteria		MetS by the 2009 harmonized criteria			0.43
		Present	Absent	Total	
	Present	143	23	166	
	Absent	70	94	164	
	Total	213	117	330	

### Factors associated with MetS among T2DM patients

Binary logistic regression analysis was done. Smoking status was not entered into the regression analysis because of the small sample size of current smokers. Variables that transferred from the bivariate analysis into the multi-variable analysis were; sex, age, marital status, occupational status, place of residence, family history for chronic disease, alcohol consumption, khat chewing, ways of spending leisure time, stress, sleep pattern, BMI, type of oil used, frequency of meal, frequency of eating fruits and vegetables, frequency of eating red meat, frequency of coffee consumption and frequency of eating processed grains. After adjusting for confounders; sex, age, BMI, frequency of meat consumption, leisure time activity, and coffee consumption had a significant association with MetS.

MetS was 2.43 times more likely to occur in females than males (AOR = 2.43; 95% CI = 1.40, 4.21). Compared to 30–39 years of age groups, the odds of MetS was 2.74, 4.22, and 4.51 times higher among 40–49, 50–59, and > = 70 years of age groups, respectively. Study participants who spent their leisure time by reading, watching TV or others sedentary activities had 2.65 times higher odds of MetS as compared to those who spent their leisure time with walking, cycling, participating in recreational sports or with doing home activities (AOR = 2.65; 95% CI = 1.47, 4.78). Patients who had coffee per day were 57% less likely to have MetS compared to those who didn’t take daily (AOR = 0.43; 95% CI = 0.21, 0.86) (Table 5).

**Table 5:** Factors associated with metabolic syndrome among type 2 diabetes mellitus patients from Dessie Referral Hospital, Amhara region, Ethiopia, 2017 (n = 330).

Variables	Categories	MetS		COR (95% CI)	P-value	AOR (95% CI)	P-value
		Yes	No				
Sex	Male	84	86	1			
	Female	112	48	2.39 (1.52,3.76)	<0.001	2.43 (1.40, 4.21)	0.002
Age	30–39	14	24	1			
	40–49	45	33	2.34 (1.05, 5.19)	0.037	2.74 (1.02, 7.37)	0.047
	50–59	66	30	3.77 (1.72,8.29)	0.001	4.22 (1.60, 11.11)	0.004
	60–69	43	36	2.05 (0.93, 4.53)	0.07	1.90 (0.71, 5.09)	0.20
	> = 70	28	11	4.36 (1.67,11.39)	0.003	4.51(1.44, 14.15)	0.01
Ways of spending leisure time	with sedentary activities	139	75	1.92 (1.21,3.04)	0.005	2.65 (1.47, 4.78)	0.001
	Walking, cycling, recreational sports or other home activity	57	59	1			
BMI	<25	88	118	1			
	> = 25	108	16	9.05 (5.0,16.40)	<0.001	9.59 (4.98, 18.47)	<0.001
coffee intake	Daily	140	117	0.36 (0.2, 0.66)	0.001	0.43 (0.21,0.86)	0.015
	Didn’t take daily	56	17	1			
Frequency of eating red meat	At least once per week	49	19	2.02 (1.13,3.62)	0.018	2.61(1.28, 5.33)	0.009
	Didn’t take	147	115	1			

Where: Sedentary activities = reading, watching television or other sedentary activities, COR = crude odds ratio, AOR = adjusted odds ratio.

## Discussion

This study was aimed to assess the prevalence of MetS and lifestyle factors for the effective prevention and management of MetS in T2DM patients. In this study, the proportion of MetS was 59.4% (53.6%, 64.5%). It was consistent with a systematic review from Sub-Saharan Africa (59.62%) and studies from Sub-Saharan Africa (60.4%), and Ghana (58%) [8, 16, 22]. Whereas it was lower than studies from Urban India (71.9%), and Kumasi, Ghana (90.6%) [23, 24]. It was also lower than studies conducted in Hawassa, South Ethiopia (70.1%), and Gonder, North-west Ethiopia (66.7%) by the same definition of MetS [9, 10]. This might be due to the differences in the level of hospital, and population characteristics. However, it was higher than studies conducted in Ho Municipality, Ghana (43.83%), and South Ethiopia (45.9%) [25, 26]. This discrepancy could be due to the differences in the study period and sample size.

The odds of MetS among females was 2.43 times more likely than males. It was similar with studies conducted in urban India, Sub-Saharan Africa, and Ethiopia [9, 10, 16, 23]. This might be because of physiological factors like menopause-related hormonal changes might lead females to metabolic impairment [12]. Moreover, females were more obese than males and less likely to do regular physical exercise [15, 27]. Further, the occupation of women was mostly sedentary. Whereas, a study from Amman, Jordan, stated that there was no significant difference in the prevalence of MetS among sex [28]. In sharp contrast to the current study findings, MetS was higher among men than women from urban North-Central Nigeria. The authors justified that the high activity profile of women might contribute to the exceptional higher prevalence of MetS among men [29].

In this study, the odds of MetS was increased with age which was supported by most studies [9, 26, 30, 31]. It is known that the level of physical activity decreases with increased age. Further, as age increases, their dependency rate may increase which exposes them to depression and the adoption of an unhealthy lifestyle. In contrast to this study, a study from rural Victoria, Australia, stated that age was negatively associated with MetS. This might be because of the death of patients at higher age groups [32].

The regression analysis showed that overweight and obesity were significantly associated with MetS. Individuals with a BMI of ≥ 25 kg/m^2^ were 9.59 times more likely to have MetS as compared to those individuals with BMI < 25kg/m^2^. It was expected and consistent with other studies [12, 22]. This might be because higher BMI worsens insulin resistance. Besides, overweight and obese individuals had higher abdominal fat which is a risk factor for high triglyceride and blood pressure.

Spending leisure time doing sedentary activities had a significant positive association with MetS. Patients who spent their leisure time watching television, reading, or other sedentary activities had 2.65 higher odds of MetS compared to those who spent walking, cycling, doing recreational sports, and doing home activities. A similar finding was reported from Australia in which increased television watching was associated with a higher prevalence of MetS [33]. Higher odds of MetS was reported from Latino and Africa Americans with higher levels of sedentary behavior [34]. This might be because sedentariness causes obesity, insulin resistance, and poor lipid metabolism.

Red meat consumption at least one times per week had a significant positive association with MetS. This is in agreement with the reports from Korea and Nepal [12, 35]. This might may be due to the contribution of high energy and saturated fat content of red meat for weight gain, insulin resistance, and high blood cholesterol level. A study also suggested that heme iron from red meat had a greater risk for MetS [36].

In this study, coffee consumption had a significant negative association with MetS. This finding was supported by the findings from the general population, even-though finding among T2DM was not accessed [37, 38]. Similarly, epidemiological reviews concluded that coffee consumption protects from MetS [39, 40]. Studies suggested that several components of coffee had antihypertensive properties [41, 42]. Another study reported that both caffeinated and decaffeinated coffee intakes were associated with a lesser risk of T2DM [43]. So, this might be due to the antihypertensive and antioxidant activity of phenolic compounds in coffee.

The finding of this study may be affected by social desirability and recall bias. So, it may induce some degree of inaccuracy in the reporting of chat chewing, smoking, alcohol consumption as well as dietary diversity patterns. In addition, inability to quantify the energy and nutrient intakes could be one of the limitations of the study.

This study concluded that the prevalence of MetS among T2DM patients was high. Being overweight and obesity, being female, increased age, not consuming coffee daily, eating red meat on a daily to weekly basis, and sedentary leisure-time activity were factors associated with MetS. So, regular screening of T2DM patients especially for those with higher ages, female, overweight and obese T2DM patients for components of MetS; and counseling of T2DM patients to do regular physical activity, consume coffee per day, control their body weight and to avoid regular red meat consumption. Further, a cohort study on the association between coffee consumption and MetS among T2DM patients is forwarded for researchers.

## Supporting information

S1 File The questionnaire used for data collection. (DOCX)

S2 File (SAV)

## Acronym/abbreviation

AOR: Adjusted Odds Ratio
BMI: Body Mass Index
BP: Blood pressure
DDS: Diet Diversity Score
dL: Deciliter
FBG: Fasting Blood Glucose
HDL: High-Density Lipoprotein
Kg: Kilogram
MetS: Metabolic Syndrome
Mg: Milligram
T2DM: Type 2 Diabetes Mellitus
TG: Triglyceride
WC: Waist Circumference

## References

[1] Kylin E. Studies of the hypertension-hyperglycemia-hyperuricemia syndrome. Zentralbl Inn Med. 1923;44:105–27.

[2] Third Report of the National Cholesterol Education Program (NCEP) Expert Panel on Detection, Evaluation, and Treatment of High Blood Cholesterol in Adults (Adult Treatment Panel III) final report. Circulation. 2002;106(25):3143–421. Epub 2002/12/18. . 12485966

[3] Grundy SM, Brewer HB Jr., Cleeman JI, Smith SC Jr., Lenfant C, American Heart A, et al Definition of metabolic syndrome: Report of the National Heart, Lung, and Blood Institute/American Heart Association conference on scientific issues related to definition. Circulation. 2004;109(3):433–8. 10.1161/01.CIR.0000111245.75752.C6 . 14744958

[4] Alberti KG, Zimmet P, Shaw J. Metabolic syndrome—a new world-wide definition. A Consensus Statement from the International Diabetes Federation. Diabetic medicine: a journal of the British Diabetic Association. 2006;23(5):469–80. Epub 2006/05/10. 10.1111/j.1464-5491.2006.01858.x . 16681555

[5] Alberti KG, Eckel RH, Grundy SM, Zimmet PZ, Cleeman JI, Donato KA, et al Harmonizing the metabolic syndrome: a joint interim statement of the International Diabetes Federation Task Force on Epidemiology and Prevention; National Heart, Lung, and Blood Institute; American Heart Association; World Heart Federation; International Atherosclerosis Society; and International Association for the Study of Obesity. Circulation. 2009;120(16):1640–5. Epub 2009/10/07. 10.1161/CIRCULATIONAHA.109.192644 . 19805654

[6] Misra A, Singhal N, Khurana L. Obesity, the Metabolic Syndrome, and Type 2 Diabetes in Developing Countries: Role of Dietary Fats and Oils. Journal of the American College of Nutrition. 2010;29(sup3):289S–301S. 10.1080/07315724.2010.10719844 20823489

[7] Saklayen MG. The Global Epidemic of the Metabolic Syndrome. Curr Hypertens Rep. 2018;20(2):12 10.1007/s11906-018-0812-z 29480368 PMC5866840

[8] Shiferaw WS, Akalu TY, Gedefaw M, Aynalem YA, Anthony D, Mengesha A, et al Metabolic syndrome among Type 2 Diabetes Patients in Sub-Saharan African countries: a systematic review and meta-analysis. 2020 10.1101/2020.05.14.20101410 32755843

[9] Bizuayehu Wube T, Mohammed Nuru M, Tesfaye Anbese A. A Comparative Prevalence Of Metabolic Syndrome Among Type 2 Diabetes Mellitus Patients In Hawassa University Comprehensive Specialized Hospital Using Four Different Diagnostic Criteria. Diabetes Metab Syndr Obes. 2019;12:1877–87. 10.2147/DMSO.S221429 31571962 PMC6756827

[10] Biadgo B, Melak T, Ambachew S, Baynes HW, Limenih MA, Jaleta KN, et al The Prevalence of Metabolic Syndrome and Its Components among Type 2 Diabetes Mellitus Patients at a Tertiary Hospital, Northwest Ethiopia. Ethiop J Health Sci. 2018;28(5):645–54. 10.4314/ejhs.v28i5.16 30607080 PMC6308785

[11] Gebremeskel GG, Berhe KK, Belay DS, Kidanu BH, Negash AI, Gebreslasse KT, et al Magnitude of metabolic syndrome and its associated factors among patients with Type 2 Diabetes mellitus in Ayder Comprehensive Specialized Hospital, Tigray, Ethiopia: a cross sectional study. BMC Res Notes. 2019;12(1):603 10.1186/s13104-019-4609-1 31533851 PMC6751785

[12] Kaur J. Assessment and screening of the risk factors in metabolic syndrome. Medical Sciences. 2014;2(3):140–52.

[13] Gami AS, Witt BJ, Howard DE, Erwin PJ, Gami LA, Somers VK, et al Metabolic syndrome and risk of incident cardiovascular events and death: a systematic review and meta-analysis of longitudinal studies. Journal of the American College of Cardiology. 2007;49(4):403–14. 10.1016/j.jacc.2006.09.032 17258085

[14] Abhayaratna S, Somaundaram N, Rajapakse H. Prevalence of the metabolic syndrome among patients with Type 2 Diabetes. Sri Lanka Journal of Diabetes Endocrinology and Metabolism. 2015;5(2).

[15] Abda E, Hamza L, Tessema F, Cheneke W. Metabolic syndrome and associated factors among outpatients of Jimma University Teaching hospital. Diabetes, metabolic syndrome and obesity: targets and therapy. 2016;9:47 10.2147/DMSO.S97561 27019600 PMC4786063

[16] Kengne AP, Limen SN, Sobngwi E, Djouogo CF, Nouedoui C. Metabolic syndrome in Type 2 Diabetes: comparative prevalence according to two sets of diagnostic criteria in sub-Saharan Africans. Diabetol Metab Syndr. 2012;4(1):22 10.1186/1758-5996-4-22 22650602 PMC3407752

[17] FAO F. Guidelines for measuring household and individual dietary diversity. Food and Agriculture Organization of the United Nations (FAO) the Food and Nutrition Technical Assistance (FANTA) Project, Rome, Italy 2007.

[18] Yahia N, Brown C, Rapley M, Chung M. Assessment of college students’ awareness and knowledge about conditions relevant to metabolic syndrome. Diabetology & metabolic syndrome. 2014;6(1):111 10.1186/1758-5996-6-111 25360161 PMC4213528

[19] Organization WH. WHO STEPS surveillance manual: the WHO STEPwise approach to chronic disease risk factor surveillance. 2005.

[20] Organization WH. Obesity: preventing and managing the global epidemic: World Health Organization; 2000. 11234459

[21] Consultation WE. Waist circumference and waist-hip ratio. Report of a WHO Expert Consultation Geneva: World Health Organization 2008:8–11.

[22] Nsiah K, Shang V, Boateng K, Mensah F. Prevalence of metabolic syndrome inType 2 Diabetes Mellitus Patients. International Journal of Applied and Basic Medical Research. 2015;5(2):133–8. 10.4103/2229-516X.157170 26097823 PMC4456889

[23] Bhatti GK, Bhadada SK, Vijayvergiya R, Mastana SS, Bhatti JS. Metabolic syndrome and risk of major coronary events among the urban diabetic patients: North Indian Diabetes and Cardiovascular Disease Study—NIDCVD-2. Journal of Diabetes and its Complications. 2016;30(1):72–8. 10.1016/j.jdiacomp.2015.07.008 26271411

[24] Agyemang-Yeboah F, Eghan BAJ, Annani-Akollor ME, Togbe E, Donkor S, Oppong Afranie B. Evaluation of metabolic syndrome and its associated risk factors in Type 2 Diabetes: a descriptive cross-sectional study at the Komfo Anokye Teaching Hospital, Kumasi, Ghana. BioMed research international. 2019;2019 10.1155/2019/4562904 31187045 PMC6521427

[25] Osei-Yeboah J, Owiredu WKBA, Norgbe GK, Yao Lokpo S, Gyamfi J, Alote Allotey E, et al The Prevalence of Metabolic Syndrome and Its Components among People with Type 2 Diabetes in the Ho Municipality, Ghana: A Cross-Sectional Study. International Journal of Chronic Diseases. 2017;2017:8765804 10.1155/2017/8765804 28293668 PMC5331170

[26] Tadewos A, Ambachew H, Assegu D. Pattern of Metabolic Syndrome in Relation to Gender among Type-II DM Patients in Hawassa University Comprehensive Specialized Hospital, Hawassa, Southern Ethiopia. Health Science Journal. 2017;11(3). 10.21767/1791-809x.1000509

[27] Alwan AH, Alhusuny A. Assessment of Metabolic Syndrome and Its Risk Factors among Patients with Type 2 DM at Merjan Teaching Hospital, Al-Hilla City.

[28] Efaishat R, Farha RA, Alefishat E. Awareness and prevalence of metabolic syndrome among high-risk individuals attending internal medicine clinics across Jordan. Tropical Journal of Pharmaceutical Research. 2016;15(9):2001–7.

[29] Puepet F, Uloko A, Akogu I, Aniekwensi E. Prevalence of the metabolic syndrome among patients with Type 2 Diabetes Mellitus in urban North-Central Nigeria. African Journal of Endocrinology and Metabolism. 2009;8(1):12–4.

[30] Fezeu L, Balkau B, Kengne A-P, Sobngwi E, Mbanya J-C. Metabolic syndrome in a sub-Saharan African setting: central obesity may be the key determinant. Atherosclerosis. 2007;193(1):70–6. 10.1016/j.atherosclerosis.2006.08.037 17011567 PMC1961628

[31] Unadike B, Akpan N, Peters E, Essien I, Essien O. Prevalence of the metabolic syndrome among patients with Type 2 Diabetes Mellitus in Uyo, Nigeria. African Journal of Endocrinology and Metabolism. 2009;8(1):9–11.

[32] Devers MC, Campbell S, Simmons D. Influence of age on the prevalence and components of the metabolic syndrome and the association with cardiovascular disease. BMJ Open Diabetes Res Care. 2016;4(1):e000195 10.1136/bmjdrc-2016-000195 27158519 PMC4853802

[33] Dunstan D, Salmon J, Owen N, Armstrong T, Zimmet P, Welborn T, et al Associations of TV viewing and physical activity with the metabolic syndrome in Australian adults. Diabetologia. 2005;48(11):2254–61. 10.1007/s00125-005-1963-4 16211373

[34] Hsu Y-W, Belcher BR, Ventura EE, Byrd-Williams CE, Weigensberg MJ, Davis JN, et al Physical activity, sedentary behavior, and the metabolic syndrome in minority youth. Medicine & Science in Sports & Exercise. 2011;43(12):2307–13. 21552153 10.1249/MSS.0b013e318222020f PMC7656775

[35] Woo HD, Shin A, Kim J. Dietary patterns of Korean adults and the prevalence of metabolic syndrome: a cross-sectional study. PloS one. 2014;9(11):e111593 10.1371/journal.pone.0111593 25365577 PMC4218781

[36] De Oliveira Otto MC, Alonso A, Lee D-H, Delclos GL, Bertoni AG, Jiang R, et al Dietary intakes of zinc and heme iron from red meat, but not from other sources, are associated with greater risk of metabolic syndrome and cardiovascular disease. The Journal of nutrition. 2012;142(3):526–33. 10.3945/jn.111.149781 22259193 PMC3278268

[37] Nordestgaard AT, Thomsen M, Nordestgaard BG. Coffee intake and risk of obesity, metabolic syndrome and Type 2 Diabetes: a Mendelian randomization study. Int J Epidemiol. 2015;44(2):551–65. 10.1093/ije/dyv083 . 26002927

[38] Shin S, Lim J, Lee HW, Kim CE, Kim SA, Lee JK, et al Association between the prevalence of metabolic syndrome and coffee consumption among Korean adults: results from the Health Examinees study. Appl Physiol Nutr Metab. 2019;44(12):1371–8. 10.1139/apnm-2018-0880 . 31663770

[39] Buscemi S, Marventano S, Antoci M, Cagnetti A, Castorina G, Galvano F, et al Coffee and metabolic impairment: an updated review of epidemiological studies. NFS Journal. 2016;3:1–7.

[40] Baspinar B, Eskici G, Ozcelik AO. How coffee affects metabolic syndrome and its components. Food Funct. 2017;8(6):2089–101. 10.1039/c7fo00388a . 28589997

[41] Zhao Y, Wang J, Ballevre O, Luo H, Zhang W. Antihypertensive effects and mechanisms of chlorogenic acids. Hypertension Research. 2012;35(4):370–4. 10.1038/hr.2011.195 22072103

[42] Geleijnse JM. Habitual coffee consumption and blood pressure: an epidemiological perspective. Vascular health and risk management. 2008;4(5):963 10.2147/vhrm.s3055 19183744 PMC2605331

[43] Ding M, Bhupathiraju SN, Chen M, van Dam RM, Hu FB. Caffeinated and decaffeinated coffee consumption and risk of Type 2 Diabetes: a systematic review and a dose-response meta-analysis. Diabetes care. 2014;37(2):569–86. 10.2337/dc13-1203 24459154 PMC3898757

