# Supplementary material for: Metabolic syndrome and lifestyle factors among type 2 diabetes mellitus patients in Dessie Referral Hospital, Amhara region, Ethiopia
Source: PLoS One. 2020 Nov 2;15(11):e0241432. doi: 10.1371/journal.pone.0241432 (PMC7605694; doi:10.1371/journal.pone.0241432)
Supplement: S1 File — (DOCX) [file pone.0241432.s001.docx]

# English Version Questionnaire

| Date of interview __________Time started________________Time completed________ Respondent code __________ Interviewer name ___________ Signature_______________ | | | |
| --- | --- | --- | --- |
| S.No | **Question**s | **Options** | **Skip** |
|  | **Part I: Socio-Demographic Information** | |  |
| 101. | Sex | 1. Male 2. Female |  |
| 102. | Age in years_________________ | |  |
| 103. | What is your religion? | 1. Orthodox 2. Muslim 3. Protestant 4. Catholic 5. other________ |  |
| 104. | What is your educational status? ______________________________ | |  |
| 105. | Marital status | 1. Single 2. Married 3. Divorced 4. Widowed |  |
| 106. | What is your occupation? ________________________________________ | |  |
| 107. | Place of residence | 1. Urban 2. Rural |  |
| 108. | Ethnicity | 1. Amhara 2. Tigre 3. Oromo 4. Afar 5. Other…. |  |
| 109. | What is your monthly per capita income? (HH income/family size) ________ | |  |
| **Part II. Medication and family history of disease** | | |  |
| 201. | Did your family have history of cardio-metabolic diseases (DM, hypertension, dyslipidemia)? | 1. Yes 2. No, if yes which one ________? |  |
| 202. | Your age at which the Diabetic Mellitus occurred (in years): _______________ | |  |
| 203. | Do you take medication for diabetes? | 1. Yes 2. No | If no skip to Q # 206 |
| 204. | If “yes” for Q no/ 203 which medication do you take | 1. tablet 2. Insulin therapy 3. both |  |
| 205. | When do you start medication? ____ month _____year | |  |
| 206. | Have you been told by a doctor or other health worker that you have any of the following? (Tick all that you have.). (Better to observe from monitoring card) | 1. Raised blood pressure or hypertension 2. High level of total cholesterol 3. Low level of HDL 4. High level of LDL 5. High level of triglyceride 6. No | If no skip to part III |
| 207. | Are you currently receiving treatments for any of the above cases prescribed by a doctor or other health worker? | 1. Yes 2. No |  |
| 208 | if yes for Q 207 describe the type of cases and treatment | |  |
| **Part III: Behavioral risk factors** | | |  |
| 301. | Did any of the adults at home smoke while you were growing up/living? | 1. Yes 2. No |  |
| 302. | Do you smoke cigarette? | 1. never smoke 2. former smoker 3. current smokers | If “A” skip to Q # 304 |
| 303. | How many cigarettes do you smoke/did you smoke per day? _____________ | |  |
| 304. | Do you chew khat? | 1. Yes 2. No |  |
| 305. | How often do you chew khat? (frequency of chewing) __________________ | |  |
| 306. | Have you drink alcohol within the past 12 months? | 1. Yes 2. No | If no skip to Q # 308 |
| 307. | Average number of drinks? _________per day or_________ per week (specify the type of drink) | |  |
|  | How many units of alcohol do you usually drink when you drink alcohol? (a beer a glass of wine, local drinks)_____________________ | |  |
| 308. | Do you do have/perform regular exercise? | 1. Yes 2. No | If no skip to part IV |
| 309. | For how many days per week_______________? | |  |
| 310. | For how many minutes per day? _________________? | |  |
| 311. | List the type of exercises that you most often perform___________________ | |  |
| 312. | How do you spend your leisure time? (tick the one that fits best) | 1. Reading, watching TV, or other sedentary activity. 2. Walking, cycling, or other forms of exercise 3. Participation in recreational sports (swimming, playing ball) heavy gardening etc 4. Other/specify______ |  |
| 313. | Do you have difficulty in initiating and maintaining sleep? | 1. Yes 2. No |  |
| 314. | For how much time do you sleep per day? __________hour | |  |
| 315. | Do you have/had stress? | 1. Yes 2. No |  |
| **Part 4. Knowledge Questions** | | |  |
| 401. | Cardio metabolic diseases (heart condition, hypertension, diabetes etc) are associated with heredity. | 1. Yes 2. No |  |
| 402. | Obese individuals have an elevated risk of heart cases than non-obese. | 1. Yes 2. No |  |
| 403. | Which one of the following is a healthy food? | 1. Processed or packed foods 2. Fried foods 3. High fat foods 4. Sweets foods 5. All 6. None |  |
| 404. | Individuals with diabetes may only eat special kinds of sweets for diabetes. | 1. Yes 2. No |  |
| 405. | Individuals with diabetes are more liable to suffer from a heart condition or stroke. | 1. Yes 2. No |  |
| 406. | Which one of the following can used to prevent complication of DM? | 1. taking drug 2. physical exercise 3. Stop smoking 4. Diversified diet 5. Maintaining healthy weight 6. All 7. None 8. Don’t know |  |
| 407. | Risk factor for cardiovascular disease | 1. Diabetes 2. Obesity 3. Hypertensions 4. Cholesterol 5. All 6. None 7. Don’t know |  |
| **Part V. Dietary Risk Factors** | | |  |
| 501. | Which meals do you eat regularly, check all that apply? | 1. Breakfast 2. Lunch 3. Dinner 4. Snack (time____) |  |
| 502. | Do you have a meal plan? | 1. Yes 2. No   If yes, please describe___ |  |
| 503. | On average over the past month, how many days per week have you followed your eating plan? | 0 1 2 3 4 5 6 7 |  |
| 504. | Which of the following eating styles you apply on a regular basis | 1. Erratic eater 2. Time constraint |  |
| 505. | What type of oil or fat is most often used for meal preparation in your household? (you can select more than 1, but the most frequent one*)* | 1. Vegetable oil 2. Palm oil 3. Suet 4. Butter 5. Other/ specify_____ |  |

|  |  | | | | | | | |
| --- | --- | --- | --- | --- | --- | --- | --- | --- |
| 506. How often do eat the following products? | >3 times per day | 2-3 times per day | once a day | Twice per week | Once a week | Once per 2 weeks | monthly | Don’t take |
| Wholegrain products (enjera, bread (teff, barley, maize, sorghum etc.)) |  |  |  |  |  |  |  |  |
| White bread |  |  |  |  |  |  |  |  |
| pasta, macaroni |  |  |  |  |  |  |  |  |
| Potato |  |  |  |  |  |  |  |  |
| Legumes (Lentils, chickpeas, /dried peas, soybeans). |  |  |  |  |  |  |  |  |
| rice |  |  |  |  |  |  |  |  |
| Sugars and sweets (cakes, cookies, chocolates, biscuits, honey, kandy) |  |  |  |  |  |  |  |  |
| Soft drinks (coca, Miranda, sprite, etc…) |  |  |  |  |  |  |  |  |
| Vegetables (Lettuce, cabbage, pepper, tomato etc.) |  |  |  |  |  |  |  |  |
| Fish |  |  |  |  |  |  |  |  |
| egg |  |  |  |  |  |  |  |  |
| Chicken |  |  |  |  |  |  |  |  |
| Red meat |  |  |  |  |  |  |  |  |
| Organ meat ( liver, heart) |  |  |  |  |  |  |  |  |
| Milk and milk products |  |  |  |  |  |  |  |  |
| Fried foods (Doughnuts, chips, Popcorn) |  |  |  |  |  |  |  |  |
| coffee |  |  |  |  |  |  |  |  |
| Tea |  |  |  |  |  |  |  |  |

507. Tell me all foods and drinks that you took yesterday

| Breakfast | Snack | Lunch | Snack | Dinner | Snack |
| --- | --- | --- | --- | --- | --- |
|  |  |  |  |  |  |

**Part VI: Anthropometric and Blood Pressure Measurements**

| **S. No** |  | Measurement 1 | measurement 2 | Measurement 3 (BP) | average |
| --- | --- | --- | --- | --- | --- |
| **601.** | Weight in Kg |  |  |  |  |
| **602.** | Height in cm |  |  |  |  |
| **603.** | WC in cm |  |  |  |  |
| **605.** | Systolic BP in mmHg |  |  |  |  |
| **606.** | Diastolic BP mmHg |  |  |  |  |

**Part VII: Result of Laboratory Diagnosis**

701. Blood glucose level ____________________ mg/dl

702. HDL_C level=__________________________ mg/dl

703. Triglyceride level=___________________________mg/dl

704. Total cholesterol =____________________________ mg/dl

# Amharic Version Questionnaire

| ቃለ መጠይቅ የተደረገበት ቀን___________________  የተጀመረበት ሰዓት ____________  ያለቀበት ሰዓት_____________________________ ኮድ____________________________________  የጠያቂው ስም_____________________________ ፊርማ ______________________ | | | |
| --- | --- | --- | --- |
| **ተ.ቁ** | **ቃለ-መጠይቅ** | **ምርጫ** | **ዝለል** |
|  | **ክፍሌ አንድ: ሥነ-ሕዝብና ኢኮኖሚን የተመለከተ ጥያቄ** | |  |
| 101. | ፆታ | ሀ. ወንድ ለ. ሴት |  |
| 102. | እድሜ _________________ | |  |
| 103. | ሃይማኖትዎ ምንድን ነው? | ሀ. ኦርቶዶክስ  ለ. ሙስሉም  ሐ. ፕሮቴስታንት  መ. ካቶሊክ  ሠ. ላሊ: ………… |  |
| 104. | የትምህርት ደረጃዎ ስንት ነው? ________________ | |  |
| 105. | የጋብቻ ሁኔታ | ሀ. ያላገባ/ች  ለ. ያገባ/ች  ሐ. የፈታ/ች  መ. የሞተበት/ባት |  |
| 106. | ስራዎ ምንድን ነው?/ በምንድን ነው የሚተዳደሩት? __________________________ | |  |
| 107. | የመኖሪያቦታ | ሀ. ከተማ ለ. ገጠር |  |
| 108. | ብሔርህ/ሽ ምንድን ነው? | ሀ. አማራ  ለ. ትግሬ  ሐ. ኦሮሞ  መ. አፋር  ሠ. ሌላ/የጠቀስ…… |  |
| 109. | በአማካኝ ወርሀዊ ገቢዎ ምን ያሕል ነው? (ጠቅላላ የቤተሰቡ ገቢ ተደምሮ ለቤተሰብ ብዛት ይካፈል፡፡) _____________________ | |  |
| **ክፍል ሁለት፡ የህከምናና የቤተሰብ ጤና ታሪክ** | | |  |
| 201. | ከዘርዎ (እናት፣አባት፣አያትወዘተ) የልብ፣የስኳር፣የደምግፊት፣የመሳሰሉ ህመሞች የታመመ አለ/ነበር? | ሀ. አዎ  ለ. የለም  አዎከሆነየትኛው? |  |
| 202. | የስኳር ሕመም የጀመረህ/ሽ በስንት አመትህ/ሽ ነው? | |  |
| 203. | የስኳርመድሀኒት ጀምረሀል/ሻል? | ሀ. አዎ  ለ. የለም | መልሱ “ለ” ከሆነ ወደ 206 ዝለል |
| 204. | ለጥያቄ 203 መልስዎ አዎ ከሆነ የሚወስዱት መድሀኒት ምንድን ነው? | 1. ኪኒን 2. መርፌ 3. ሁለቱም |  |
| 205. | መቼነው መድሀኒት የጀመሩት? ወር ______ አመተምህረት ________ | |  |
| 206. | በዶክተር ወይም በሌለላ ጤና ባለሙያ ከሚከተሉት አለብዎት የተባሉት የትኛውን ነው?ያለባቸው ሁሉም ይመረጥ (ከካርዳቸው ታይቶይሞላ) | ሀ. የደምግፊት  ለ. የጠቅላላ ኮለስትሮል  ሐ. የኤችዲኤል ኮለስትሮል  መ. የትራይግላይሰሪድ  ሠ. የለም | መልሱ “የለም” ከሆነወደክፍል 3 ዝለል |
| 207. | በጥያቄ 206 ለተዘረዘሩት አሁን የታዘዘልዎት የሚወስዱት መድሃኒት አለ? | ሀ. አዎ  ለ. የለም |  |
| 208. | ለጥያቄ 207 መልስዎ አዎ ከሆነ ለየትኛው ህመም እንደሆነ ይገለጽ፡፡ | |  |
| **ከፍል 3፡ባህሪን የተመለከተ ጥያቄ** | | |  |
| 301. | እርስዎ ባደጉበት /በሚኖሩበት ቤት ውስጥ ሲጋራ የሚያጨስ ነበር? | ሀ. አዎ  ለ. የለም |  |
| 302. | ሲጋራ ታጨሳለህ/ሽ? | ሀ. አጭሸአላውቅም  ለ. በፊትአጨስነበር  ሐ. አጨሳለሁ | መልሱ ሀ; ከሆነ ወደ 304 ዝለል |
| 303. | በቀን ስንት ሲጋራ ታጨሳለህ/ሽ/ ነበር?­­­­­­­ | |  |
| 304. | ጫት ትቅማለህ/ሽ? | ሀ. አዎ  ለ. የለም |  |
| 305. | በየስንት ጊዜነው የምትቅመው/ሚው? | |  |
| 306. | ባለፈው 12 ወርውስጥአልኮልጠጥተሀል/ሻል? | ሀ. አዎ  ለ. የለም | መልሱ “ለ” ከሆነ ወደ 308 ዝለል |
| 307. | በአማካኝ በቀን/ በሳምንት ስንት ትጠጣለህ/ሽ? ( የመጠጡአይነትይገለፅ) በቀን___________/በሳምንት______________________ በአንድ ጊዜ ስንት ይጠጣሉ? | |  |
| 308. | ስፖርት ትሰራለህ/ሽ? | ሀ. አዎ  ለ. የለም | መልሱ “ለ” ከሆነወደ 312 ዝለል |
| 309. | በሳምንት ስንት ቀን? _______________ | |  |
| 310. | ለስንት ደቂቃ? _________________ | |  |
| 311. | የምትሰራቸው/ሪያቸው የሰፖርት አይነቶች ምንምን ናቸው? | |  |
| 312. | ትርፍ ጊዜዎትን እንዴት ያሳልፈሉ? | ሀ. ማንበብ፣ፊልም (ተቪ) ማየት፣እናሌላቁጭተብለውየሚታለፉ  ለ. ወክ፣ሳይክልመንዳት፣የመሳሰሉ  ሐ. የመዝናኛሰፖርቶችበመስራት  ሠ. ሌላ/ ይጠቀስ-------- |  |
| 313. | የእንቅልፍ ችግር አለብዎት (አልወስድ ይልዎታል)? | ሀ. አዎ  ለ. የለም |  |
| 314. | ስንት ሰዓት በእንቅልፍ ያሳልፋሉ( በአንድ ቀን ለስንት ሰዓት ይተኛሉ)? | |  |
| 315 | ጭንቀት አለብዎት? | ሀ. አዎ  ለ. የለም |  |
| **ክፍል 4. እውቀትን የተመለከተ ጥያቄ**  **ቄ** | | | |
| 401. | የስኳር፣የደምግፊት፣የልብ ህመም በዘር ይወረሳል፡፡ | ሀ. እውነት  ለ. ሐሰት |  |
| 402. | ወፍራም ሰዎች ካለወፈሩት የበለተ ለልብ ሕመም ይጋጣሉ፡፡ | ሀ. እውነት  ለ. ሐሰት |  |
| 403. | ከሚከተሉት ውስጥ ጤናማ ምግብ ያለሆነውን ምረጥ፡፡ | ሀ. የታሸጉ ምግቦች  ለ. የተጠበሱ ምግቦች  ሐ. ከፍተኛ ስብ/ቅባት ያላቸው ምግቦች  መ. ጣፋጭ ምግቦች  ሠ. ሁሉም ጤናማ ምግቦች ዐይደሉም  ረ. መልስ የለም |  |
| 404. | የስኳር ሕመም ያለባቸው ሰዎች የተለየ አይነት ጣፋጭ መውሰድ አለባቸው፡፡ | ሀ. እውነት  ለ. ሐሰት |  |
| 405. | ስኳር ያለባቸው ሰዎች ለልብ ሕመም የመጋለጥ አቅማቸው ከፍተኛ ነው፡፡ | ሀ. እውነት  ለ. ሐሰት |  |
| 406. | የስኳር ሕመምተኞች ተጨማሪ ሕመሞችን ለመከላከል ምን ማደረግ አለባቸው? | ሀ. መድሀኒት በአግባቡ መውሰድ  ለ. እንቅስቃሴ/ ስፖርት መስራት)  ሐ. ሲጋራ አለማጨስ  መ. የተመጣጠነ ምግብ መመገብ  ሠ. ክብደትን መቆጣጠር  ረ. ሁሉም  ሰ. መልስ አልተሰጠም  ሸ. አላውቅም |  |
| 407. | ለልብ ህመም የሚያጋለጠው የትኛው ነው? | ሀ. የስኳር ሕመም  ለ. ውፍረት  ሐ. የደምግፊት  መ. ኮለሰትሮል  ሠ. ሁሉም  ሰ. መልስ አልተሰጠም  ሸ. አላውቅም |  |
| **ክፍል 5. አመጋገብን የተመለከተ ጥያቄ*(ለፍስክ ምግቦች ፆም ከመጀመሩ በፉት ያለውን ጊዜ ይውሰዱ)*** | | |  |
| 501. | በቋሚነት የሚመገቡት የትኛውን ነው? (ከአንድ በላይ መምረጥ ይቻላል) | ሀ. ቁርስ  ለ. ምሳ  ሐ. ራት  መ. መክሰስ(ጊዜ--------) |  |
| 502. | የምግብ ዕቅድ/ሜኑ አለዎት? | ሀ. አዎ  ለ. የለም  መልስዎአዎከሆነይገለጽ--------- | መልሱ “ለ” ከሆነወደ 504 ዝለል |
| 503. | በአማካኝ ምን ያህል ቀን/ናት በሳምንት ውስጥ ይህን የአመጋገብ እቅድዎን ይከተላሉ? | 0 1 2 3 4 5 6 7 |  |
| 504. | ብዙጊዜ የምትከተሉት የአመጋገብ ሁኔታ የትኛው ነው? | ሀ. ያልተስተካከለ አመጋገብ (ሰዓት ጠብቄ አልመገብም)  ለ. ሰአት ጠብቆ ተመጋቢ |  |
| 505. | ብዙጊዜ ምግብለ ማዘጋጀት የምትጠቀሙት ዘይት/ቅባት ምንድንነው? | ሀ. የአትክል ትዘይት  ለ. የሚረጋ ረዘይት  ሐ. ሞራ  መ. ቅቤ  ሠ. ሌላ/ይጠቀስ-------- |  |

| **506. ከዚህ በታች የተዘረዘሩትን ምግቦች የአመጋገብዎ ድግግሞሽ እንዴትነው?** | በቀን ከ 3 ጊዜ በላይ | በቀንከ 2-3 ጊዜ | በቀን 1 ጊዜ | በሳምንት 2 ጊዜ | በሳምንት 1 ጊዜ | በ 2 ሳምንት 1 ጊዜ | በወር  1ጊዜ | በ6 ወር 1 ጊዜ | በአመት 1 ጊዜ | ጭራሽ አልወስድም |
| --- | --- | --- | --- | --- | --- | --- | --- | --- | --- | --- |
| የእህልዘር (እንጀራ፣ዳቦ (የጤፍ፣ገብስ፣ስንዴ፣ማሽላ፣በቆሎ ወዘተ)) |  |  |  |  |  |  |  |  |  |  |
| የፍርኖ ዳቦ |  |  |  |  |  |  |  |  |  |  |
| ፓስታ፣መኮረኒ፣ |  |  |  |  |  |  |  |  |  |  |
| ድንች |  |  |  |  |  |  |  |  |  |  |
| የጥራጥሬ ዘር ( ባቄላ፣ምስር፣አተር፣ሽምብራ) |  |  |  |  |  |  |  |  |  |  |
| ሩዝ |  |  |  |  |  |  |  |  |  |  |
| ስኳር እና ጣፋጮች (ኬክ፣ኩኪስ፣ብስኩት፣ከረሜላ) |  |  |  |  |  |  |  |  |  |  |
| ለስላሳ መጠጦች |  |  |  |  |  |  |  |  |  |  |
| የታሸጉ ጁሶች |  |  |  |  |  |  |  |  |  |  |
| ፍራፍሬ ( ሙዝ፣ብርቱካን፣ፓፓያ፣ማንጎ፣አቮካዶ) |  |  |  |  |  |  |  |  |  |  |
| አትክልቶች (ጎመን፣ሰላታ፣ቆስጣቲማቲም፣ቃሪያወዘተ) |  |  |  |  |  |  |  |  |  |  |
| ዓሳ |  |  |  |  |  |  |  |  |  |  |
| እንቁላል |  |  |  |  |  |  |  |  |  |  |
| የዶሮሥጋ |  |  |  |  |  |  |  |  |  |  |
| ቀይሥጋ ( የከብት፣የፍዬል፣የበግ) |  |  |  |  |  |  |  |  |  |  |
| የኦርጋን ስጋ ( ጉበት፣ልብኩላለት) |  |  |  |  |  |  |  |  |  |  |
| ወተትና የወተት ተዋፅዖ |  |  |  |  |  |  |  |  |  |  |
| የተጠበሱ ምግቦች( ችብስ፣ሳምቡሳ፣ቦንቦሊኖ፣ዶናት፣ፈንዲሻየመሳሰሉ) |  |  |  |  |  |  |  |  |  |  |
| ቡና |  |  |  |  |  |  |  |  |  |  |
| ሻይ |  |  |  |  |  |  |  |  |  |  |

**ትላንትበ 24 ሰአት ውስጥ የተመገቡት ምግብና መጠጦች መመዝገቢ ሰንጠረዥ**

| **ቁርስ** | **ከቁርስ በኋላ** | **ምሳ** | **መክሰስ** | **እራት** | **ከራት በኋላ** |
| --- | --- | --- | --- | --- | --- |
|  |  |  |  |  |  |

**ክፍል 6: የሰውነት እና የደም ግፊት ልክ**

| ተ.ቁ |  | ልክ 1 | ልክ 2 | ልክ 3 (ለደምግፊት) | አማካኝ |
| --- | --- | --- | --- | --- | --- |
| 601. | ክብደት በኪ.ግ |  |  |  |  |
| 602. | ቁመት በሴ.ሜ |  |  |  |  |
| 603. | ወገብ በሴ.ሜ |  |  |  |  |
| 605. | ሲያስቶሊክ የደምግፊት በሚ.ሜ ሜርኩሪ |  |  |  |  |
| 606. | ዲያሰቶሊክ የደም ግፊት በሚ.ሜ ሜርኩሪ |  |  |  |  |

**ክፍል 7: የላብራቶሪ ውጤት**

701. የስኳር መጠን_______________________ mg/dl

702. የኤችዲኤል ኮለስትሮል መጠን =_____________________________mg/dl

703. የትራይግላይሰሪድ መጠን=__________________________mg/dl

704. የጠቅላላ ኮለስትሮል መጠን =__________________________
